# Scaling Up International Food Aid: Food Delivery Alone Cannot Solve the Malnutrition Crisis

**DOI:** 10.1371/journal.pmed.0050235

**Published:** 2008-11-25

**Authors:** The PLoS Medicine Editors

## Abstract

The*PLoS Medicine* Editors discuss what role food aid should play in addressing the global childhood malnutrition crisis.

In January 2008, in an article entitled “The Starvelings,” *The Economist* made the case that childhood hunger and malnutrition have a far greater impact upon child health than was previously thought [1]. The most recent estimates are that stunting, severe wasting, and intrauterine growth restriction together are responsible for 21% of disability-adjusted life years and 2.2 million deaths in children under five years annually [2]. Those malnourished children who do survive are at heightened risk of long-term health, educational, and economic impairment [3]. Hunger is now arguably “the gravest single threat to the world's public health” [1].

There was therefore a tremendous sense of urgency among participants of the recent two-day international meeting in New York City on preventing and treating childhood malnutrition, organized by Columbia University's Institute of Human Nutrition and the humanitarian organization Médecins sans Frontières (MSF). Christophe Fournier, President of MSF's International Council, called childhood malnutrition an “invisible crisis” and asked: “Why are we witnessing countless deaths from a preventable cause?”

There is no simple answer. UNICEF's framework on the determinants of nutritional status includes a complicated array of interconnected “basic determinants”—ecological, economic, and sociopolitical [4]. These basic determinants in turn give rise to three specific “underlying determinants”: the quality of health care services, caregiver resources such as maternal knowledge about child care, and food security (defined as secure access for all people at all times to sufficient quality and quantity of food in order to lead a healthy and active life [5]).

An important challenge in preventing malnutrition is determining which of these three basic determinants should be the priority target. This is a highly contentious issue within the child nutrition community, and there does not seem to be a consensus. Some nutrition experts and health agencies, for example, believe that the focus should be on boosting caregiver resources—particularly educating mothers to feed their children the right foods [6,7]. The World Bank argues that most serious malnutrition is caused by bad sanitation and disease, leading to diarrhea, especially among young children, and that “women's status and women's education play big parts in improving nutrition” [7].

The child hunger crisis includes a knowledge crisis.

In contrast, the Columbia University meeting focused on only one of the three determinants—food insecurity, particularly in crisis regions where many humanitarian agencies work. Several speakers at the meeting argued that the world's poor lack access to food and that improving health services or educating mothers alone will not be enough to address the malnutrition crisis. A study by Save the Children UK, for example, conducted in Bangladesh, Myanmar, Ethiopia, and Tanzania, found that between 15% (in Ethiopia) and 79% (in Bangladesh) of households were simply too poor to adequately feed their children a healthy diet [5]. A systematic review by Dewey and Adu-Afarwuah found that educating mothers alone has little effect on childhood growth, morbidity, or development in regions where household food insecurity is prevalent, unless mothers also have access to nutrient-rich foods [8].

The meeting did not discuss or examine long-term solutions to food insecurity. Such solutions would include an array of policies to stimulate local agricultural and economic development (particularly the economic and social empowerment of women, the primary caregivers in most households) so that communities become food-secure in a sustainable and independent way. Instead, the meeting explored how the international community can urgently deliver more food aid, of better quality, to prevent and treat malnutrition in the highest-burden areas. Such an emergency measure is clearly needed to bring down death rates as quickly as possible—but it is not a sufficient long-term approach to the global malnutrition crisis.

In examining the role of food aid in preventing malnutrition, the meeting's focus was on improving access to nutrient-rich complementary (weaning) foods from the ages of six months to two years, a critical window for child growth. Standard cereal-based food aid, used in many maternal and child health programs, usually consists of corn or wheat soy blends, which are mixed with water and served as a gruel [9]. Are these vegan fortified blends adequate for preventing malnutrition? This is another contentious issue. Few studies have evaluated the effect of feeding these blends to children at risk of malnutrition, and their efficacy in large-scale nutrition programs is not well established [10–12].

One concern about these blends is that they contain “anti-nutrients” that may interfere with nutrient absorption and dietary fibers that can affect appetite [9]. Another concern, which MSF is heavily publicizing, is whether the lack of animal-source foods in these blends is contributing to nutritional deficiencies and growth retardation. To support this view, MSF points to a 1994 study showing nutritional deficiencies and growth retardation in Dutch children aged four to 18 months who had been fed a macrobiotic vegan diet [13]. But several child nutrition experts that we spoke to say that this Dutch study may not be applicable to the use of corn or wheat soy blends in child feeding programs—unlike macrobiotic diets, these food blends are fortified. Nevertheless, one of the key take-home messages from the Columbia meeting was that food supplements for complementary feeding should not only be energy dense, nutrient rich, and free of anti-nutrients, but they should also contain animal-source foods such as milk. MSF has recently adopted a policy of including at least some animal-source food in its nutrition programs. “We would not provide our own children only cereal porridge, why do we accept a double standard in food aid?” said Tido von Schoen-Angerer, Executive Director of MSF's Access to Essential Medicines Campaign.

MSF is now promoting a complementary food supplement that it believes has the right composition: ready-to-use supplementary food. This supplement is typically a peanut-based paste enriched with milk solids, vitamins, and nutrients—it has a long shelf life and, unlike cereal-based food aid, it does not need to be mixed with water [14]. The idea of using such ready-to-use supplements for preventing malnutrition comes from the success of ready-to-use therapeutic food in the community-based treatment of severe acute malnutrition, which is the most life-threatening of all types of malnutrition. Based on solid clinical trial evidence, this treatment approach has now been endorsed by the World Health Organization, the World Food Programme, UNICEF, and the UN System Standing Committee on Nutrition [15]. Could a ready-to-use supplement also *prevent* malnutrition in high-burden areas (the 25–30 countries where the vast majority of children under three years old are at risk)?

Donor-supported food programs are not enough as a long-term strategy.

Although the idea remains highly controversial, there is support for it from preliminary efficacy trials. For example, Patel and colleagues compared standard food aid (corn soy blend) with ready-to-use food as complementary feeding regimens in children at risk of malnutrition from seven centers in rural Malawi [16]. Children who received ready-to-use food had greater rates of weight gain than children who received corn soy blend. In another trial [17], 313 Ghanaian infants were randomized to one of three complementary food supplements from the age of six to 12 months: (1) a ready-to-use food called Nutributter (a peanut-based spread that includes milk powder); (2) a multiple micronutrient powder called Sprinkles (the efficacy of this powder in controlling childhood anemia has been previously presented in *PLoS Medicine* [18]); and (3) a crushable multiple micronutrient tablet called Nutritabs. The Nutributter group had greater length and weight gains than did the other two supplementation groups. But skeptics say that there is not yet enough scientific evidence to support scaling up of ready-to-use supplementary food as a preventive intervention. As Martin Enserink recently reported in his news feature in *Science*: “The issue has pitted those who want to see solid evidence before embarking on a major aid program against those—impatient with talk about *P*-values, cost-effectiveness, and sustainability—who want to act now” [14].

Steve Collins, one of the founders of Valid International, which works to improve the delivery of humanitarian assistance, said at the meeting that in the malnutrition crisis we face “a problem, an opportunity, and a threat.” The problem is that malnutrition is the world's greatest cause of underdevelopment and poverty, its scale is huge, and it is inextricably linked with other global problems including the HIV pandemic, global warming, and the food and population crises. The opportunity is that the G8 has begun talking about nutrition [19], and community-based treatment of severe acute malnutrition with ready-to-use therapeutic food has created a “positive vibe” in the nutrition community [15]. But the threat is that there is a lack of clarity in discussions about childhood malnutrition, its prevention, and its treatment. This lack of clarity was on show at the Columbia University meeting—in discussions about ready-to-use foods, for example, it was hard to tell whether speakers were talking about their role in prevention or in treatment, and it was hard to know which stage of malnutrition was being discussed. Different diets, said Collins, will be needed for different types of malnutrition, and a variety of food products need to be developed for targeted food assistance. With all these products there will be questions about their efficacy, delivery, cost, and sustainability.

The unfortunate reality is that the child hunger crisis includes a knowledge crisis—we still have scientific gaps, for example, in our understanding of what would be the perfect complementary food supplement for food aid. “It is sobering to realize,” wrote Lutter and Dewey, “that far more is known about formulating optimal foods for domestic livestock than about complementary feeding of young children” [20]. And while developing better quality complementary food supplements clearly has an important role to play in addressing the malnutrition crisis and saving lives now, particularly in emergency situations, donor-supported food programs are not enough as a long-term strategy.

Other proven malnutrition interventions must also be scaled up. For example, the Ending Child Hunger and Undernutrition Initiative, a global partnership started by UNICEF and the World Food Programme, is calling for the global scale-up of a range of “practical measures” [21]. These include health, hygiene, and nutrition education and promotion, micronutrient supplementation, household water treatment, hand washing, deworming, and “situation-specific household food security interventions” [21]. National and international development strategies and policies, together with political will and financing, are also required [22]. It will cost about $US8 billion a year to assist 100 million families to protect their children from hunger and malnutrition [21]—and yet current donor spending on programs to reduce undernutrition is only about $US250–$US300 million annually [23].

We commend the humanitarian aid agencies such as MSF and Valid International for, as one child health expert told us, “shaking up the ossified systems of international food aid.” Food aid alone, however, as Lautze and colleagues have noted, “cannot be sufficient for combating the multi-faceted nature of the current [malnutrition] emergency” [24]. The reactive approach to dealing with malnutrition, although appropriate in responding to humanitarian crises, risks creating long-term dependency on medicalized food aid. Addressing the role of food aid in long-term efforts will require answering some difficult questions, including which basic determinants of malnutrition should be the major focus and who should coordinate the global response to the malnutrition crisis. The dialogue must therefore extend beyond the immediate context of food delivery to the broader sociopolitical sphere, including the role of humanitarian agencies and nongovernmental organizations themselves. We will explore many of these concerns in an upcoming commissioned series in *PLoS Medicine*. We invite you to join the debate by sending Reader Responses to the series.

## Abbreviations

MSF: Médecins sans Frontières
